# Biomechanical comparison of different interspinous process devices in the treatment of lumbar spinal stenosis: a finite element analysis

**DOI:** 10.1186/s12891-022-05543-y

**Published:** 2022-06-17

**Authors:** Zhengpeng Liu, Shuyi Zhang, Jia Li, Hai Tang

**Affiliations:** 1 Department of Orthopaedics, Beijing Friendship Hospital, Capital Medical University, Xicheng District, Beijing, 101100 China; 2 Department of Spine Surgery, Affiliated Hospital of Chengde Medical University, Chengde, 067000 Hebei China; 3 Department of Joint Surgery, Affiliated Hospital of Chengde Medical University, Chengde, 067000 Hebei China

**Keywords:** Lumbar spinal stenosis, Finite element analysis, Biomechanics, Interspinous process devices, BacFuse, Coflex, X-Stop

## Abstract

**Background:**

Lumbar spinal stenosis (LSS) is a common disease among elderly individuals, and surgery is an effective treatment. The development of minimally invasive surgical techniques, such as the lumbar interspinous process device (IPD), has provided patients with more surgical options.

**Objective:**

To investigate the biomechanical properties of different IPDs, including BacFuse, X-Stop and Coflex, in the treatment of LSS.

**Methods:**

Based on the computed tomography images of a patient with LSS, four finite element (FE) models of L3-S5 were created in this study. The FE models included a surgical model of the intact lumbar spine and surgical models of the lumbar IPDs BacFuse, X-Stop, and Coflex. After validating the models, they were simulated for four physiological motions: flexion, extension, lateral bending and axial rotation, and range of motion (ROM). Stress distribution of discs and facet joints in each segment, stress distribution of the spinous process in the operated section, and stress distribution of the internal fixation were compared and analysed.

**Results:**

Compared to the model of the intact lumbar spine, the other three models showed a decrease in ROM and disc and facet joint stresses in the surgical segment during movement and an increase in ROM and disc and facet joint stresses in the adjacent segments. These effects were greater for the proximal adjacent segment with BacFuse and more pronounced for the distal adjacent segment with Coflex, while X-Stop had the greatest stress effect on the spinous process in the surgical segment.

**Conclusion:**

BacFuse, Coflex and X-Stop could all be implemented to effectively reduce extension and disc and facet joint stresses, but they also increase the ROM and disc and facet joint stresses in adjacent segments, which may cause degeneration.

## Background

Lumbar spinal stenosis (LSS) is defined as the narrowing of the spinal canal in the lower part of the back and the compression of the dura vesicle, spinal cord or nerve roots, resulting in corresponding neurological deficits that can cause pain or numbness in the lower limbs and intermittent claudication. The culprits of the disease are bulging discs, hyperplasia of the facet joints or ligament flava hypertrophy. The most common cause of LSS is degenerative spondylosis, which commonly affects the elderly population [1]. LSS, which fails to respond to conservative treatment, usually requires surgical intervention. The most common surgical treatment is single- or multisegment laminectomy for decompression, in which tissues such as osteophytes and ligament flava are removed to expand the volume of the spinal canal, which can relieve chronic pain and enable patients to resume daily activities quickly.

As minimally invasive surgery is widely used, a growing number of surgeons are adopting lumbar interspinous process devices (IPDs) to treat patients with LSS. Kong et al. [2] reported 1-year follow-up results after Coflex implantation and traditional fusion for patients with degenerative spinal stenosis and showed that the clinical outcome of the CoflexTM group was similar to that of the PLIF group. Despite the limitations of that study in terms of the length of follow-up period, CoflexTM reduced ROM at the instrumented level and may not affect ROM as much as PLIF in the upper adjacent motion segment. The authors also believed that implantation of CoflexTM may be an alternative or better treatment for patients with instable degenerative lumbar spinal stenosis under certain conditions. However, in a randomised controlled trial using an IPD and conventional surgery, Moojen et al. [3] were unable to confirm the hypothetical short-term advantage of the interspinous process device over conventional simple decompression, and even showed a fairly high reoperation rate after implantation of an IPD. In a comparison of minimally invasive decompression with the X-Stop, Lønne et al. [4] found that the procedure cost was significantly increased, as was the risk of secondary surgery, and in a meta-analysis of 27 studies (2241 patients), Mo et al. [5] showed that when compared to lumbar fusion, the X-Stop system produced no significant improvements in the Oswestry disability index (ODI), visual analogue scale (VAS) or Japanese Orthopaedic Association (JOA) scores or disc height.

Controversies persist with regard to the study of IPDs [6], as they still possess a great deal of value and varied designs; their advantages and disadvantages are equally controversial. IPDs indirectly decompress the nerve root via implantation into the interspinous process of the responsible segment to limit the extension of the lumbar spine, expand the aperture of the nerve hole and maintain the partial ROM of the motor unit to alleviate the patient’s symptoms. If the patient’s symptoms cannot be relieved by buckling, then traditional decompression surgery is required. Therefore, the IPD is designed as an internal stabilizer that reduces facet joint stress, expands the intervertebral foramen and releases the compressed lumbar root nerve [7].

IPDs are usually implanted in patients with single- or double-segment LSS at L1-L5 and can be used in multiple segments. Insertion of an IPD requires a smaller incision and fewer muscle contractions, contributing to a shorter procedure time and less blood loss. However, it is controversial whether the results of IPD insertion are superior to those of bony decompression alone [6]. However, considering the advantages of this minimally invasive surgery, most patients still choose IPD implantation.

Less commonly reported is the new IPD BacFuse, which is also a minimally invasive spinal procedure that preserves the supraspinal ligament and relies on rivets between the pterygoid plates on either side for fixation of the spine. The US Food and Drug Administration (FDA) classifies BacFuse as an internal spinal fixation system for degenerative diseases of the lumbar spine, such as lumbar disc herniation (LDH) and LSS. According to previous clinical studies [8, 9], it was reported that BacFuse could reduce postoperative pain, improve lumbar function, increase posterior disk height and foraminal height, and reduce morbidity and recurrence rates. However, there are no comparative biomechanical studies available, and there is a need to assess how different design features of IPD affect lumbar spine biomechanics.

In this study, FE modelling of the intact L3-S5 lumbar spine was developed based on CT data of the lumbar spine in patients with LSS, and three IPDs, BacFuse, Coflex and X-Stop, were fitted to the L4-L5 spinous process based on interspinous height to simulate the postoperative outcomes of the three IPDs. After validation of the model, the ROM, disc stresses, facet joint stresses and spine stress distribution in the surgical segments and adjacent segments were compared with the intact lumbar spine.

## Methods

### FE modelling of the lumbar spine

In this experiment, a volunteer with LSS was selected. He was diagnosed with LSS (L4-5, central type) after enquiring about his medical history and conduction of a physical examination and imaging. The patient showed intermittent claudication of both lower limbs and needed to rest after walking approximately 50 m, which was relieved by bending over and resting, while regular conservative treatment, such as medication and physiotherapy, was ineffective, with recurrent symptoms. The patient’s preoperative thin layers of lumbosacral vertebrae computed tomography (CT) data (0.5 mm) were stored separately on a compact disc read-only memory (CD-ROM) via the picture archiving and communication system (PACS) of the institution after a review of the imaging and medical records had ruled out certain spinal diseases (e.g., scoliosis, spondylolysis, lumbar space-occupying diseases and lumbar tuberculosis).

The lumbar spine images from the CT scan were imported into three-dimensional reconstruction software Mimics 20.0 (Materialise, Belgium) in Digital Imaging and Communications in Medicine (DICOM) format. After the processing of mask masking, a 3D model of the lumbosacral spine was generated and exported in STL, which was then imported into Geomagic Warp 19.0 (Geomagic, USA), and the 3D skeletal model was smoothed, denoised and reverse-engineered to create a 3D geometric model of the L3-S5 vertebral body consisting of cortical bone, cancellous bone and posterior structures, with 1 mm of cortical bone [10]. The graphical data were saved in the Initial Graphics Exchange Specification (IGES) format.

Next, the IGES file was imported into SolidWorks2021 (Dassault, France), and the lumbar spine sequence was adjusted according to the postoperative X-ray results to create the L3-4, L4-5 and L5-Sl intervertebral discs according to the lumbar spine anatomy, including the superior and inferior cartilage endplates, the nucleus pulposus and the anulus fibrosus, which has a 0.5 mm endplate [11, 12], and the nucleus pulposus volume makes up approximately 35% of the intact disc [13]. The vertebral body and disc model were saved in an x-t file for backup.

The x-t file was imported into Ansys Workbench 21.0 (Ansys, USA), and the ligamentous tissues of the anterior longitudinal ligament (ALL), posterior longitudinal ligament (PLL), ligamentum flava (LF), capsular ligament (CL), supraspinous ligament (SSL), interspinous ligament (ISL) and intertransverse process ligament (ITL) were created according to the anatomy of the lumbar spine. Muscle tissue is covered in this model. The final complete FE modelling of the lumbar spine includes the L3-S5 vertebral body, the L3-Sl disc tissue, the facet joints on both sides and various ligaments. In view of the degree of degeneration in patients with LSS, the nucleus pulposus of the mildly degenerative disc was changed from liquid to solid properties, and the elastic modulus was set at twice that of the normal intervertebral disc anulus fibrosus matrix, with reference to the study by Natarajan [14]. The material properties of each part are shown in Table 1 [15].

**Table 1:** Material properties

Component	Young’s modulus (MPa)	Poisson ratio	Cross-sectional area (mm2)
Cortical bone	12,000	0.3	
Cancellous bone	100	0.2	
Posterior bone	3500	0.25	
Endplate	3000	0.25	
Articular cartilage	25	0.4	
Titanium alloy	110,000	0.3	
Normal Nucleus pulposus	4.2	0.45	
Normal Annulus fibrosus	1	0.499	
Mild degeneration Nucleus pulposus	4.2	0.45	
Mild degeneration Nucleus pulposus	8.4	0.45	
Anterior longitudinal ligament (ALL)	20	0.3	40
Posterior longitudinal ligament (PLL)	20	0.3	20
Ligament flava (LF)	19.5	0.3	40
Supraspinous ligament (SSL)	15	0.3	40
Interspinous ligament (ISL)	12	0.3	30
Intertransverse ligamen (ITL)	59	0.3	10
Capsular ligament (CL)	32.9	0.3	30

### FE modelling of the surgical procedures

**Fig. 1:**
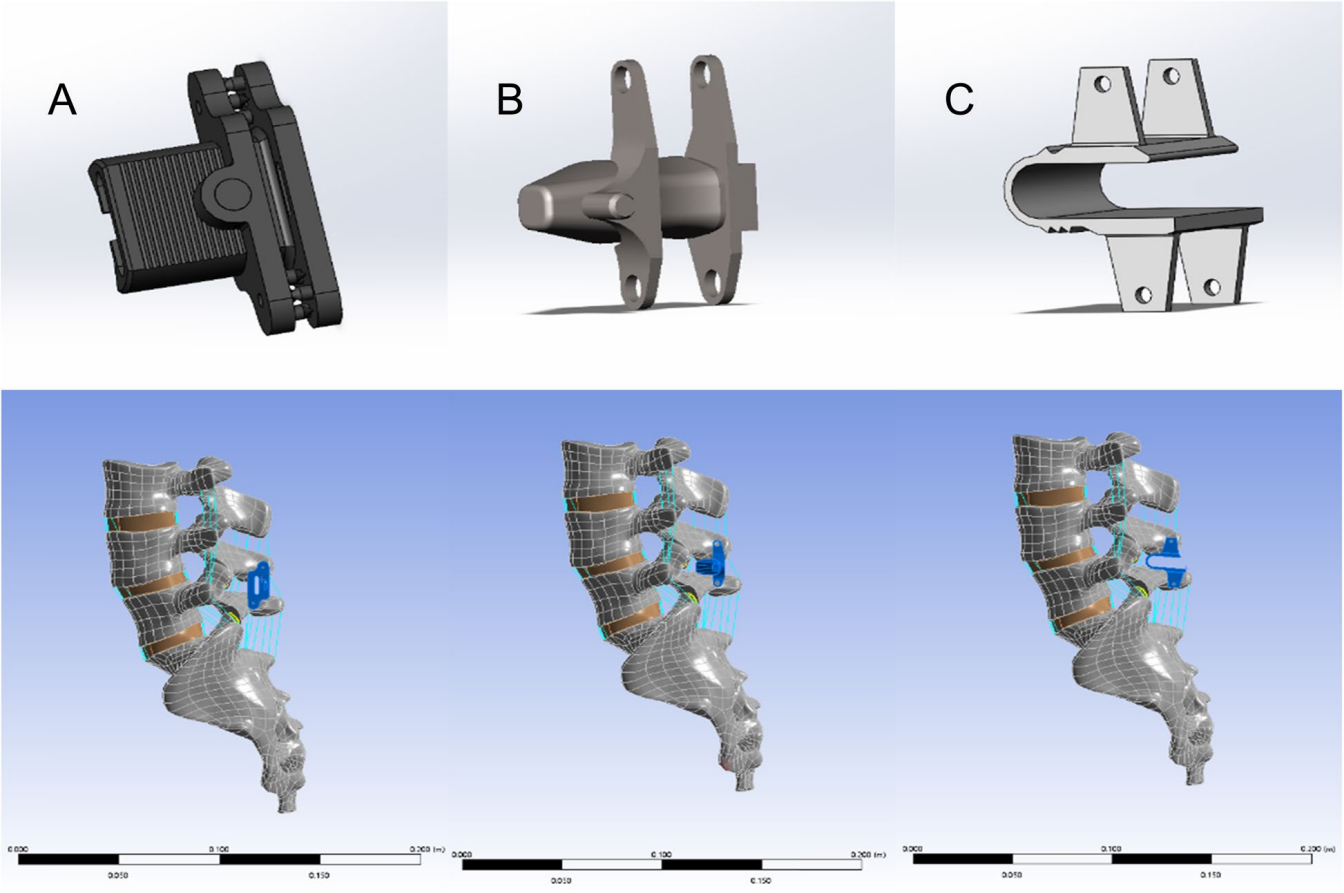
Interspinous process device (IPD) models. **A** BacFuse **B** X-Stop **C** Coflex

Based on the manufacturer’s drawings and actual measurements of the internal fixation, SolidWorks 2021 was used to construct 3D models of the IPDs, BacFuse, X-Stop and Coflex, with the appropriate product size selected based on the patient’s interspinous height and spinous process morphology. In the intact lumbar FE model, BacFuse, X-Stop and Coflex were simulated in the L4-L5 segment according to the operating manual (Fig. 1). A 0.1 frictional contact factor was used between the facet joints in the setup [16]. The intact lumbar spine model and the internal fixation model were meshed with a 2 mm mesh and unit forms of hexahedral and tetrahedral elements.

### Boundary and loading conditions

Based on previous studies [17], a hybrid testing protocol was used to simulate the effect of IPD on the surgical segment and adjacent segments. There were two cases: 1) for model validation, all degrees of freedom below the S1 vertebral segment were fixed, and a 10 Nm pure moment was applied to the upper L3 endplate; 2) for model comparison, all degrees of freedom below the S1 vertebral segment were fixed, a 400 N axial load was applied to the upper L3 endplate, as well as moments in different directions, and the moment was gradually increased until the total ROM of the model was similar to that of the intact model. The deviation of each model was controlled to within 0.5°. Four physiological motions of the lumbar spine, flexion, extension, lateral bending and axial rotation, were simulated, and ROM values, facet joint stresses, spinous process stresses and intervertebral disc stresses were recorded.

## Results

### Model validation

**Fig. 2:**
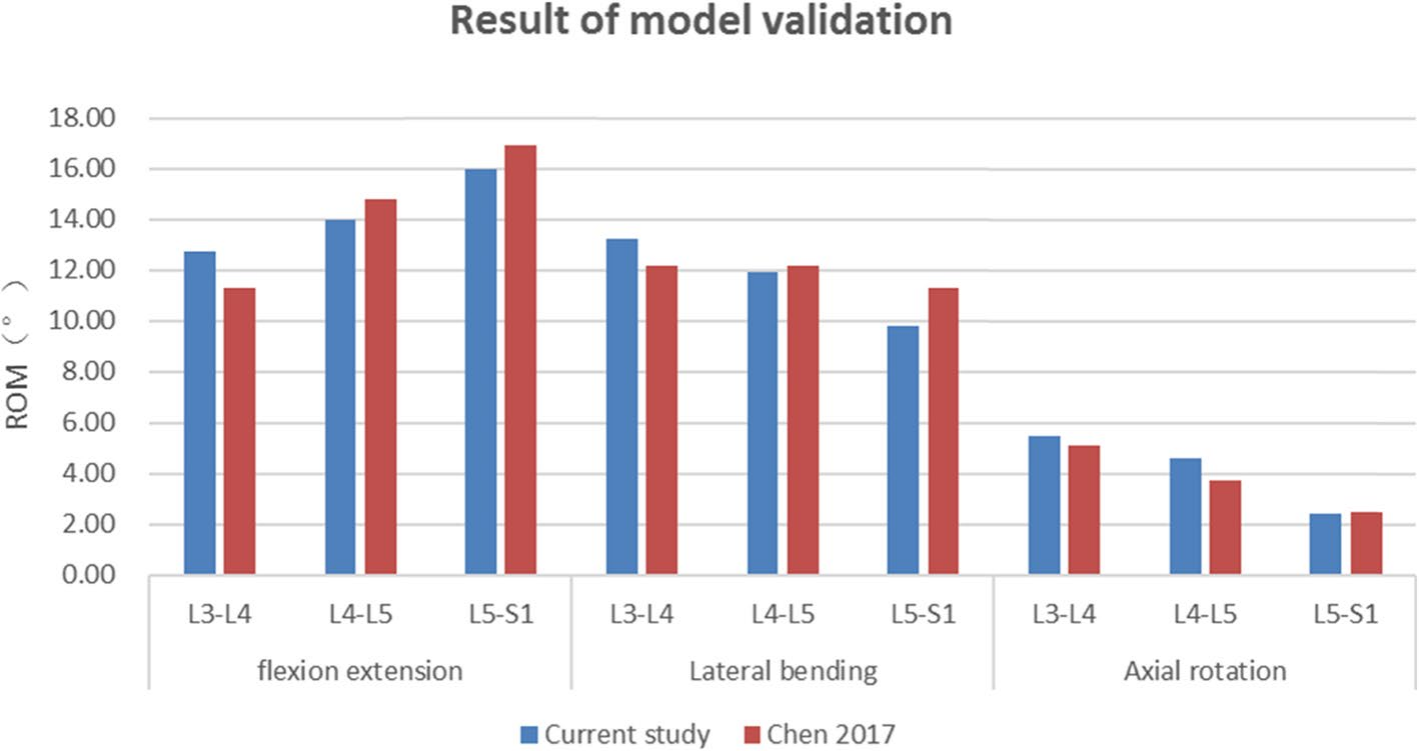
Comparison of the intersegmental ROM between the current intact model and the outcomes from previous publications

The intersegmental ROMs of the intact FE model are in accordance with those of a previous publication (Fig. 2) [18], suggesting that the intact L3-S5 FE model in the present study was successfully constructed and could be used for further modelling and analysis.

### ROMs of the IPD segments and adjacent segments

**Fig. 3:**
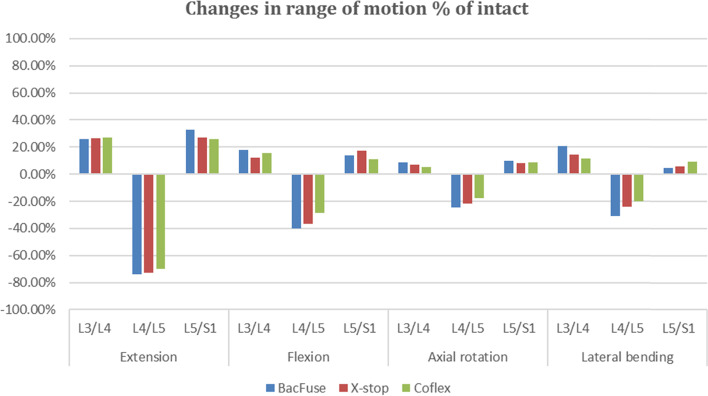
Difference in ROM as a percentage of the intact model (percent of intact) at the implanted and adjacent levels in extension, flexion, axial rotation and lateral bending

Compared to the intact model, extension was significantly restricted in all three devices implanted at L4-L5, BacFuse (-74.23 percent), X-Stop (-72.81 percent) and Coflex (-70.21 percent). There was a corresponding increase in motion in adjacent segments, with an average 26 percent in the proximal adjacent segment and a 32.73 percent increase in the distal adjacent segment, with BacFuse showing the greatest increase (shown in Fig. 3).

In flexion, the ROM of the three IPD segments all decreased to varying degrees: BacFuse (- 40.10 percent), X-Stop (-36.88 percent) and Coflex (-28.47 percent). In flexion, the ROM of the distal and proximal adjacent segments increased to varying degrees, between 11 and 18 percent, with BacFuse showing the greatest increase and a consistent trend across models (shown in Fig. 3).

In axial rotation, there was the largest decrease in BacFuse (-24.39 percent) and the smallest decrease in Coflex (-17.65 percent) compared to the intact model. There was an increase for each model in ROM in adjacent segments, but the difference in change in effect was not significant, all less than 10 percent.

In lateral bending, the BacFuse segment demonstrated the largest decrease in ROM (-30.92 percent) compared to the intact model and an increase in ROM for the adjacent segments in all models, with less impact in the distal adjacent segment and more change in the proximal adjacent segment, with a 20.51 percent change in ROM for BacFuse (shown in Fig. 3).

### Intervertebral disc stress in the IPD segment and adjacent segment

**Fig. 4:**
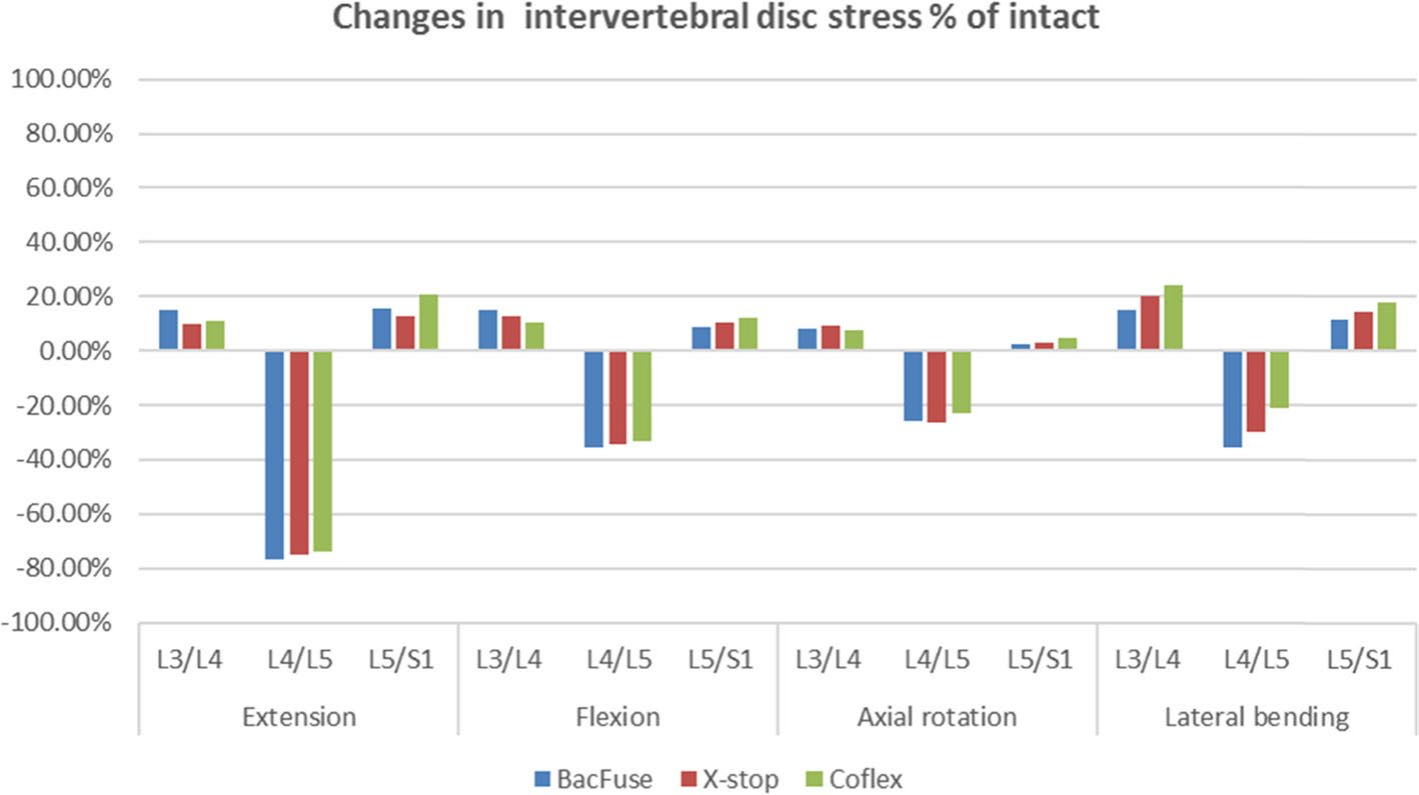
Differences in intervertebral disc stress as a percentage of the intact model (percent of intact) at the implanted and adjacent levels in extension, flexion, axial rotation and lateral bending

Figure 4 shows the maximum intervertebral disc stress at the fixation level and adjacent segments for the BacFuse, X-Stop and Coflex models. The peak intervertebral disc stresses at all IPD segments decreased by more than 70% compared to the intact model during extension. In BacFuse, distal and proximal intervertebral disc stresses increased by approximately 15%. Peak stresses at the level near L3-L4 increased by 10.06 percent in X-Stop; Coflex had a greater effect on distal disc stresses, increasing disc stresses at the L5-S1 segment by approximately 20.63 percent (Fig. 4).

In flexion, the intervertebral disc stresses at the surgical segment in each model decreased by approximately 34% in the IPD segments, with BacFuse having a greater effect on the superior segment and Coflex having a greater effect on the inferior segment with respect to the adjacent segmental disc stresses (Fig. 4).

In axial rotation, BacFuse showed the greatest reduction in intervertebral disc stress in the IPD segment, approximately 1/4, with no significant change in adjacent segments in any of the models (Fig. 4).

In lateral bending, BacFuse showed the greatest reduction in intervertebral disc stress at the IPD segment (35.41%), followed by X-Stop (29.87%) and Coflex (21.37%). Disc stresses increased in adjacent segments in each model, with Coflex showing the greatest change, with a 24.09 percent increase in the proximal adjacent segment and an 18.14 percent increase in the distal adjacent segment, and a smaller change in the BacFuse model, with 1.76 percent and 14.80 percent distally and proximally, respectively (shown in Fig. 3).

### Spinous process stress in the IPD segment

In extension, L4 and L5 spine process stresses increased in all models, with L4 spine stresses being greatest in the X-Stop model and L5 spine stresses being greatest in the Coflex model.

In flexion, lateral bending and axial rotation, L4 spinous process stresses decreased while L5 spinous process stresses increased, with the most significant decrease in the L4 segment in the BacFuse model and the most significant increase in the L5 segment in the X-Stop model, while BacFuse had the least effect on spinous process stresses in the L5 segment.

### Facet joint stress in the IPD segment and adjacent segments

**Fig. 5:**
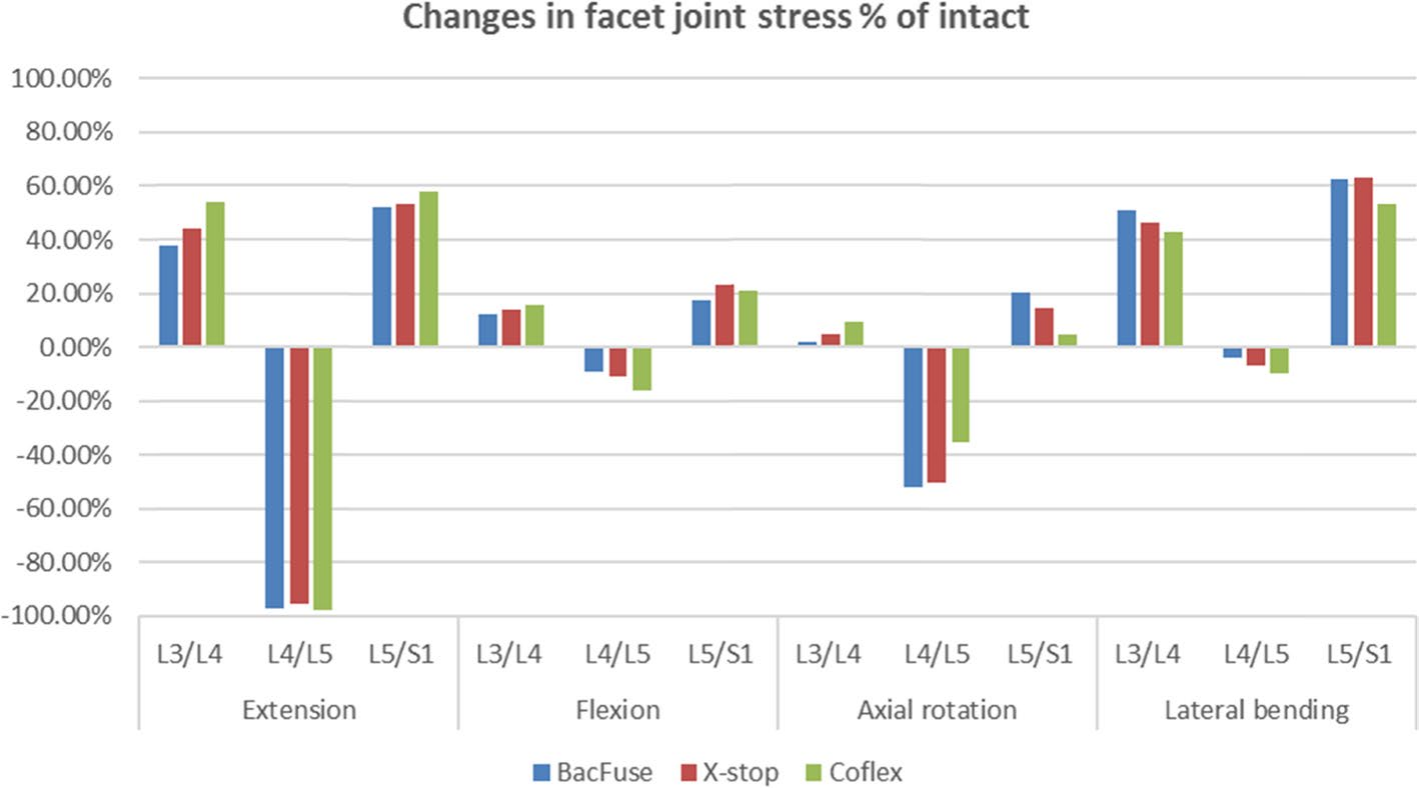
Difference in facet joint stress as a percentage of the intact model (percent of intact) at the implanted and adjacent levels in extension, flexion, axial rotation and lateral bending

Figure 5 shows the bilateral facet joint loads in the IPD segment and adjacent segment during movement. The articular surface contact forces in the IPD segment during extension are lower than those in the intact model, but the forces are greater in the adjacent segment. Compared to the intact model in flexion, all segments of the IPD-implanted model had significantly reduced facet joint stresses by approximately 67 percent, with an increase of approximately 12–15 percent proximally and 17–28 percent distally, with BacFuse having a smaller effect on the adjacent segments. The Coflex model showed the least reduction in IPD segments during rotation, with BacFuse and X-Stop being the same, and the proximal adjacent segment increase being less than 10 percent, with the distal adjacent segment increase being more pronounced in BacFuse, which increased by 20.6 percent. In lateral bending, there was less change in IPD segment a but a significant increase in all adjacent segments, with the greatest change in BacFuse (Fig. 5).

### IPD stress

**Fig. 6:**
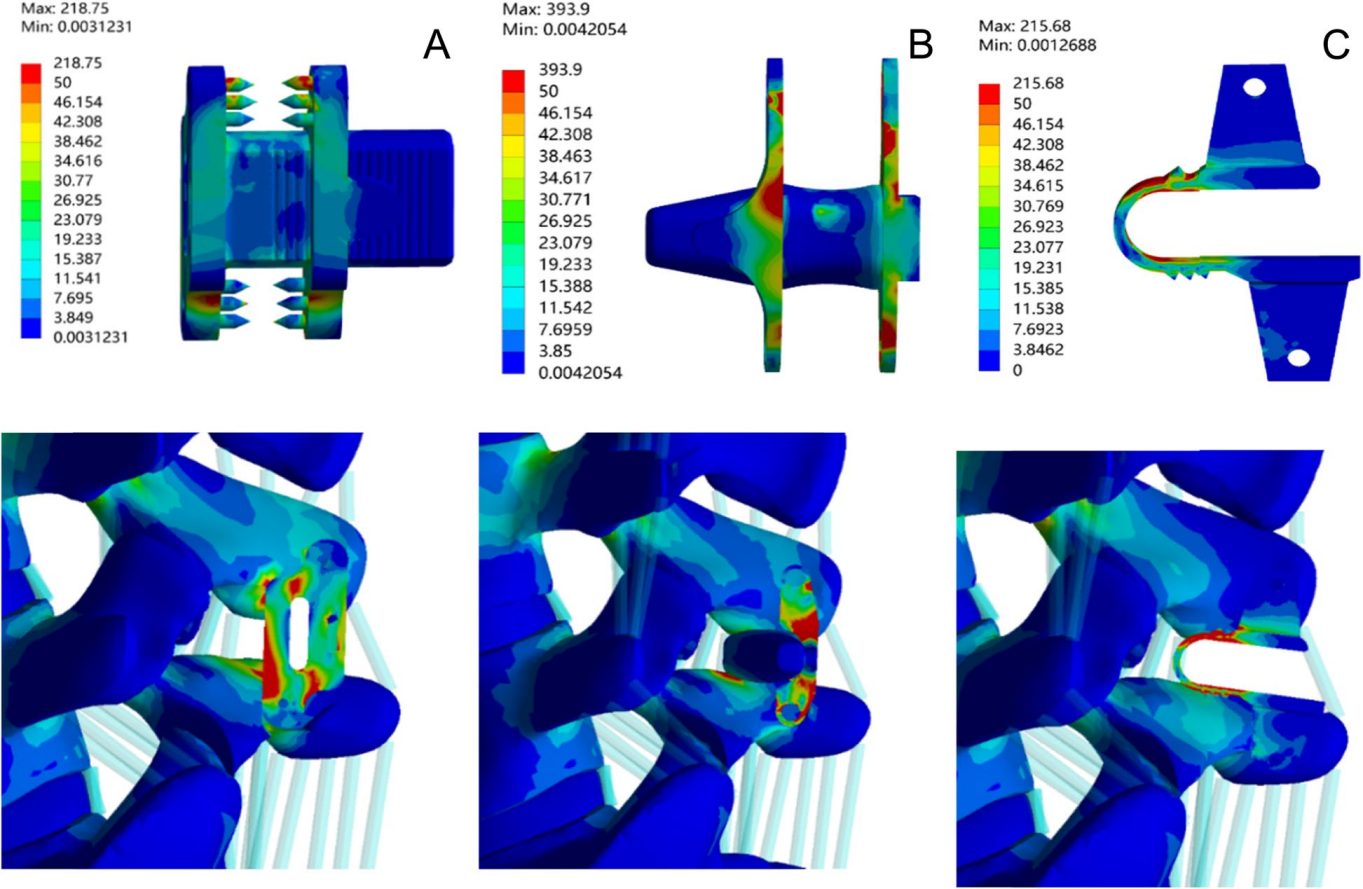
Stress distribution on the IPD in extension. **A** BacFuse **B** X-Stop **C** Coflex

Considering that the internal fixation is under the most stress during extension of the lumbar spine, Fig. 6 shows the stress distribution of the internal fixation and spinous process in each model during extension. The results show that the three fixators have different modes of force transfer. BacFuse stress distribution concentrated on the part of the fixation needle that contacts the spinous process anteriorly under the instrumentation, the X-Stop stress distribution concentrated on the fixed wings on both sides, and the Coflex stress distribution concentrated on its U-shaped part. In their peak stress results, BacFuse was close to Coflex and much less than X-Stop, at 218.74 MPa, 215.684 MPa and 393.904 MPa, respectively.

## Discussion

The design principle of IPD is to implant a fixation device with limited trauma to apply support between the spinous processes of the spinal segments for indirect decompression of the spinal canal and nerve root canal to limit lumbar extension and, to varying degrees, movements such as flexion and lateral bending and rotation, ultimately preserving some mobility of the spinal motor units. This approach is particularly suitable for elderly patients who have a variety of underlying conditions that make lumbar decompression and fusion under conventional general anaesthesia risky and have more complications. Many clinical studies have confirmed that IPDs can, to some extent, relieve some symptoms, such as low back pain and lower limb neurological function, with low risk.

Previous studies [19] have shown that Coflex can significantly inhibit the IPD segments in extension and lateral flexion ROM values but has no significant effect on adjacent segments. Several other studies [20] have concluded that single-segment implantation of the Coflex, when loaded in different directions, does not fully compensate for spinal instability. A meta-analysis including 27 clinical studies and 2241 patients [5] concluded that the ROM of the three IPDs was significantly greater in the surgical segment compared to posterior lumbar interbody fusion (PLIF), but there were no significant differences between the three spinous devices. In this study, the BacFuse, X-Stop and Coflex models had comparable effects on ROM, intervertebral disc stress and facet joint stress during extension in the IPD segment, with little difference, and they all achieved the design objective of reducing extension in the IPD segment and reducing stress on the intervertebral disc and facet joints. Among the subtle differences, the BacFuse model has the least extension ROM, and Coflex has the greatest extension in the IPD segment, which indicates that the BacFuse is more restrictive on extension. Implants increased the ROM of adjacent segments and may lead to greater pressure on the back of the intervertebral disc, which is consistent with the study of LoHJ [21] and others. BacFuse has the greatest restriction on both lateral bending and axial rotation, which is related to the way the three devices are fixed in contact with the spinous process and the shape and material of the implant. BacFuse is an internal spinal fixation device that contacts the spinous process surface and the spinous process root with uniform forces and tends to be mechanically stable, while Coflex contacts the entire upper and lower spinous process, and X-Stop only contacts the spinous root, both of which are dynamic fixation devices that tend to preserve motion.

The effect on the adjacent segmental intervertebral discs and facet joints was similar for all three devices in extension, with a significant increase in stress, especially in the facet joints, which is related to the fact that the IPD increases posterior stiffness and assumes most of the forces transmitted downwards through the facet joint, so there is essentially no significant deformation of the operation segment, which reduces the disc and facet joint stresses at the level of fixation, while the main stresses are transmitted downwards through the spinous process and facet joint, thus creating significant stresses on the posterior structure and increasing facet joint stresses. The increased stresses on the adjacent segmental facet joints may lead to accelerated degeneration and the possible development of new facet joint-related back pain.

Whitesides TE [22] applied eight cadaveric specimens to study the L2-L5 segments and placed X-Stop between the L3-L4 spinous processes, which significantly reduced their fibrous annulus and intervertebral disc pressure. This result is consistent with our study. In flexion, disc pressures decreased by approximately 1/3 in the BacFuse, X-Stop and Coflex models of the surgical segment, with less effect on disc pressures in the adjacent segments. This is most likely because the implant was designed primarily to restrict extension with less constraint on flexion.

In axial rotation, all three models reduced intervertebral disc stresses in the surgical segment, but the BacFuse was fixed firmly to the spinous process of the surgical segment. The fixation method of the BacFuse was to hold the spinous process of the surgical segment firmly in place, and this fixation resulted in higher stiffness of the surgical segment and greater stresses on adjacent segments during axial rotation. These results are consistent with the cadaveric study by Tsai and others [23].

Our results show that the BacFuse model has the greatest change in intervertebral disc stress in the surgical segment and the least change in the adjacent segment during lateral bending, which indicates that BacFuse has the least effect on disc stress in the adjacent segment. In general, these results are similar to those of spinal fusion studies [24, 25] and are more consistent with the BacFuse ‘fusion’ design concept.

Previous studies have suggested that the greatest facet joint contact forces occur during extension [20], and Wiseman [26] used seven cadaveric specimens to measure L2-L5 facet joint pressures after implanting X-Stop in the L3-L4 segment. X-Stop results in a significant decrease in small joint pressure in the L3-L4 segment, with no significant change in the adjacent L2-L3 segment. All three IPD models implanted in this study showed a significant reduction in facet joint stresses on the segments, but all adjacent segments showed an increase in facet joint stresses, with the increase being more pronounced in the distal segments. While both Coflex and BacFuse provided excellent resistance to extension, BacFuse showed a smaller change in joint stress over adjacent segments. This is largely dependent on the differences in fixation methods between these devices. The Coflex provides elastic support to the posterior column of the spine with greater deformation, whereas the BacFuse and X-Stop provide more rigid support with less deformation. An FE study by Byun and others [19] showed a 170% average increase in facet joint contraction forces in the upper adjacent segment with the Coflex implant, but values for lower adjacent segments were not recorded. One possible reason for the increase in adjacent facet joint forces after insertion of the IPD is the posterior shift of the transient rotation axis towards the implant, resulting in an increase in forces transmitted through the posterior column of the adjacent articular surfaces. The results of this study suggest that IPD implantation leads to an increase in adjacent facet joint forces, which may lead to an increased risk of adjacent facet joint hypertrophy.

IPD can cause several surgical complications, such as spinous fracture, device fracture and dislocation [6, 27], with spinous fracture being the main complication of IPD [5]. This is because the placement of the IPD during flexion and extension shifts the stress mechanism on the spinous process from tension to pressure, resulting in elevated spinous stresses and an increased risk of spinous fracture and fatigue fracture of the implant. A cadaveric study by Shepherd and others [28] documented an average breaking load of 339 N on the intact spinous process under upwards loading of the spinous device. Some authors have also optimized the structure of Coflex by means of topological optimization to reduce the concentration of spinous stress and reduce the incidence of complications [29, 30]. The results of our study showed that the maximum spinous stresses occurred during extension and that the maximum contact forces on the spinous process occurred at the L4 spinous process in all fixation models, with the highest in the X-Stop model, which is consistent with the clinical findings of spinous fractures reported in a systematic review [31], where up to 14 spinous fractures cases were reported in the X-Stop, significantly higher than the 5 cases in the Coflex, with the difference being statistically significant. It is possible that the spine disruption load may be reduced if under repeated loading, leading to the occurrence of spine fractures. This is one of the limitations of this study. In the multicentre study by Gazzeri and others [32], the postoperative sphenoid fracture rate after implantation of various IPDs was approximately 2.05% on average. However, patients treated with titanium X-Stop alone had the highest risk of fracture, with an incidence rate of 3.79% [33, 34].

This study has several limitations stemming from the simplified FE model. All vertebral bodies were simplified into sections of cortical bone, cancellous bone and posterior structures. Although this was not true for every vertebral body, the material properties of all vertebral bodies were also considered to be homogeneous and isotropic. Furthermore, the loading conditions are not identical to physiological loading, as these FE models cannot be simulated as true muscle contractions [35].

## Conclusion

BacFuse, Coflex and X-Stop can all be designed to effectively reduce extension ROM and reduce disc and facet joint stresses in the operative segment. The effect of BacFuse on adjacent segments is closer to the effect of pedicle screw fixation, which needs further comparative studies. Coflex significantly limits the ROM of the operation segment, with less change in ROM of adjacent segments but increased disc and facet joint stresses, which may accelerate degeneration of adjacent segments. X-Stop has the greatest stress on the spinous process, which is likely to cause spinous fracture. Given its effect on adjacent segments, clinical indications need to be strictly controlled and used with caution.

## Data Availability

The datasets used and/or analysed during the current study are available from the corresponding author on reasonable request. Readers can access the data and material supporting the conclusions of the study by contacting Zhengpeng Liu at 980,820,710@qq.com.

## Abbreviations

LSS: Lumbar spinal stenosis
IPD: Interspinous process device
CT: Computed tomography
DICOM: Digital Imaging and Communications in Medicine format
CD-ROM: Compact disc read-only memory
ALL: Anterior longitudinal ligament
PLL: Posterior longitudinal ligament
LF: Ligamentum flava
CL: Capsular ligament
SSL: Supraspinous ligament
ISL: Interspinous ligament
ITL: Intertransverse process ligament
FE: Finite element
PLIF: Posterior lumbar interbody fusion
ROM: Range of motion
